# Accuracy of artificial intelligence model for infectious keratitis classification: a systematic review and meta-analysis

**DOI:** 10.3389/fpubh.2023.1239231

**Published:** 2023-11-24

**Authors:** Randy Sarayar, Yeni Dwi Lestari, Arnaud A. A. Setio, Ratna Sitompul

**Affiliations:** ^1^Residency Program in Ophthalmology Faculty of Medicine, Universitas Indonesia, Jakarta, Indonesia; ^2^Department of Ophthalmology, Faculty of Medicine Universitas Indonesia – Cipto Mangunkusumo General Hospital, Jakarta, Indonesia; ^3^Digital Technology and Innovation, Siemens Healthineers, Erlangen, Germany

**Keywords:** deep learning, artificial intelligence, infectious keratitis, systematic review, accuracy

## Abstract

**Background:**

Infectious keratitis (IK) is a sight-threatening condition requiring immediate definite treatment. The need for prompt treatment heavily depends on timely diagnosis. The diagnosis of IK, however, is challenged by the drawbacks of the current “gold standard.” The poorly differentiated clinical features, the possibility of low microbial culture yield, and the duration for culture are the culprits of delayed IK treatment. Deep learning (DL) is a recent artificial intelligence (AI) advancement that has been demonstrated to be highly promising in making automated diagnosis in IK with high accuracy. However, its exact accuracy is not yet elucidated. This article is the first systematic review and meta-analysis that aims to assess the accuracy of available DL models to correctly classify IK based on etiology compared to the current gold standards.

**Methods:**

A systematic search was carried out in PubMed, Google Scholars, Proquest, ScienceDirect, Cochrane and Scopus. The used keywords are: “Keratitis,” “Corneal ulcer,” “Corneal diseases,” “Corneal lesions,” “Artificial intelligence,” “Deep learning,” and “Machine learning.” Studies including slit lamp photography of the cornea and validity study on DL performance were considered. The primary outcomes reviewed were the accuracy and classification capability of the AI machine learning/DL algorithm. We analyzed the extracted data with the MetaXL 5.2 Software.

**Results:**

A total of eleven articles from 2002 to 2022 were included with a total dataset of 34,070 images. All studies used convolutional neural networks (CNNs), with ResNet and DenseNet models being the most used models across studies. Most AI models outperform the human counterparts with a pooled area under the curve (AUC) of 0.851 and accuracy of 96.6% in differentiating IK vs. non-IK and pooled AUC 0.895 and accuracy of 64.38% for classifying bacterial keratitis (BK) vs. fungal keratitis (FK).

**Conclusion:**

This study demonstrated that DL algorithms have high potential in diagnosing and classifying IK with accuracy that, if not better, is comparable to trained corneal experts. However, various factors, such as the unique architecture of DL model, the problem with overfitting, image quality of the datasets, and the complex nature of IK itself, still hamper the universal applicability of DL in daily clinical practice.

## 1 Introduction

Keratitis is a sight-threatening condition that requires rapid and accurate management to prevent irreversible outcomes. Among all etiologies, infectious keratitis (IK) is the most common cause to corneal blindness, with an estimated incidence ranging from 2.5 to 799 per 100,000 population-year (1). Globally, it is among the top five leading causes of vision impairment and blindness, accounting for 1.5–2.0 million cases of blindness yearly (2). IK can be caused by extensive range of microbial organisms, including bacteria, fungi, viruses, parasites and polymicrobial infections. It was once considered as a ‘silent epidemic’ in low-income and middle-income countries (LMICs) with an incidence of 113–799 per 100,000 populations per year compared to high-income countries (HICs) with an incidence rate of 2.5–4.3 per 100,000 populations per year (1, 3, 4). It is very highly likely that occupational factors, such as agricultural work, environmental factors, and abuse of traditional eyedrops or eye wash contribute to the high incidence number in LMICs. These factors precipitated by limited access to primary and secondary care consequently leads to the patients to suffer from intractable infection and at higher risk of losing eyesight (1, 5, 6). Moreover, as IK usually affects patients in their productive years, it has magnified the financial burden of both individuals and the countries affected.

It is critical to note that the outcome of IK depends heavily on prompt treatment following a timely and accurate diagnosis. Current practice of IK diagnosis that involves utilizing slit lamp photography of infectious cornea disease among cornea specialists has been reported to only yield 66.0–75.9% of accuracy in differentiating bacterial and fungal keratitis (7). Corneal scraping and biopsy come as the ‘gold standard’ method for definitive IK diagnosis. However, these approaches are oftentimes dampened by the challenges in LMICSs where there may be a lack of access ophthalmic units with clinical experts and standardized investigating equipment, resulting in reliance in empirical treatment and delay in definitive treatment. Moreover, even when the option of microbiological workups is available, this procedure might also take days before producing any results. These challenges may be held accountable for poorer clinical outcome and higher risk of irreversible sequalae. On the other hand, poorly differentiated clinical feature may also lead to misdiagnosis, which may result in disastrous cascade of inappropriate treatment, increasing the risk of unidentified clinically essential lesions (8), making human decision-making for diagnosis even more difficult.

Artificial intelligence (AI) has captured the attention of medical specialties, such as ophthalmology, dermatology, and radiology, where visual analysis and interpretation skill are highly demanded to create diagnosis. Deep learning (DL), a subfield of AI, has shown promising potential in assisting automated clinical diagnosis and decision-making by performing high-dimensional analyzes, henceforth improving healthcare efficiency. The ophthalmology field is not a stranger to DL itself, with previous studies have reported diagnostic accuracy that, if not better, is comparable to clinical experts in making the diagnosis of posterior segment diseases, including macular degeneration, glaucoma, and diabetic retinopathy (9–14). One DL-based technology has also been reported with remarkable sensitivity and specificity, with the respective number of 91.3 and 91.1% in detecting vision-threatening diabetic retinopathy (14). Apart from its accuracy, availability of DL may also enable clinician to provide better care without needing to accommodate resources in constant purchasing, maintaining, training, and upgrading expensive capital for diagnostic laboratory equipment and technicians. Furthermore, it may also assist ophthalmologists and even empower untrained clinicians to diagnose IK in resource-scarce regions and take the needed actions, therefore diminishing its progressivity to debilitating corneal-related blindness. Despite its promising potential and the number of studies demonstrating high accuracy of DL in IK diagnosis and recognizing IK apart from other ocular diseases (8, 15–20), its diagnostic accuracy remains to be elucidated.

To the best of our knowledge, there is no published systematic review and/or meta-analysis on this topic to this date. Accordingly, we conducted a comprehensive systematic review and meta-analysis aiming to evaluate the accuracy of the available artificial intelligence algorithms to correctly classify infectious keratitis based on the etiology compared to its respective gold standard, i.e., the human counterparts. It is expected that this systematic review and meta-analysis may be a strong basis for the consideration of DL deployment into clinical practice, therefore preventing delay and providing better care for IK patients.

## 2 Materials and methods

### 2.1 Search strategy

Literature was searched in six online scientific databases (PubMed, Google Scholar, ProQuest, ScienceDirect, Cochrane, and SCOPUS) on December 10^th^, 2022. Search phrases include a combination of all main keywords and their related terms: “artificial intelligence” and “keratitis,” as described in Table 1. Reference lists of each study were also analyzed for potentially relevant articles. Identification of new studies via other methods was not performed. There was no restriction in the publication year.

**Table 1:** Online databases used and the respective search strategy.

No.	Database	Search terms
1	PubMed^®^	[Keratitis OR “corneal ulcer” OR “corneal diseases” OR “corneal lesion”(MeSH Terms)] AND [“Artificial intelligence” OR “machine learning” OR “deep learning”(MeSH Terms)]
2	Google scholars	(Keratitis OR “corneal ulcer” OR “corneal diseases” OR “corneal lesion”) and (“artificial intelligence” or “deep learning” or “machine learning”)
3	Proquest^®^	mainsubject (keratitis OR “corneal ulcer” OR “corneal diseases” OR “corneal lesion”) AND mainsubject (“artificial intelligence” OR “deep learning” OR “machine learning”)
4	ScienceDirect^®^	(Keratitis OR “corneal ulcer” OR “corneal diseases” OR “corneal lesion”) and (“artificial intelligence” or “deep learning” or “machine learning”)
5	Cochrane^®^	(Keratitis OR “corneal ulcer” OR “corneal diseases” OR “corneal lesion”) in All Text AND (“artificial intelligence” or “deep learning” or “machine learning”) in All Text - (Word variations have been searched)
6	Scopus^®^	[TITLE-ABS-KEY (keratitis OR “corneal ulcer” OR “corneal diseases” OR “corneal lesion”) AND TITLE-ABS-KEY (“artificial intelligence” OR “deep learning” OR “machine learning”)]

### 2.2 Study selection

**Figure 1:**
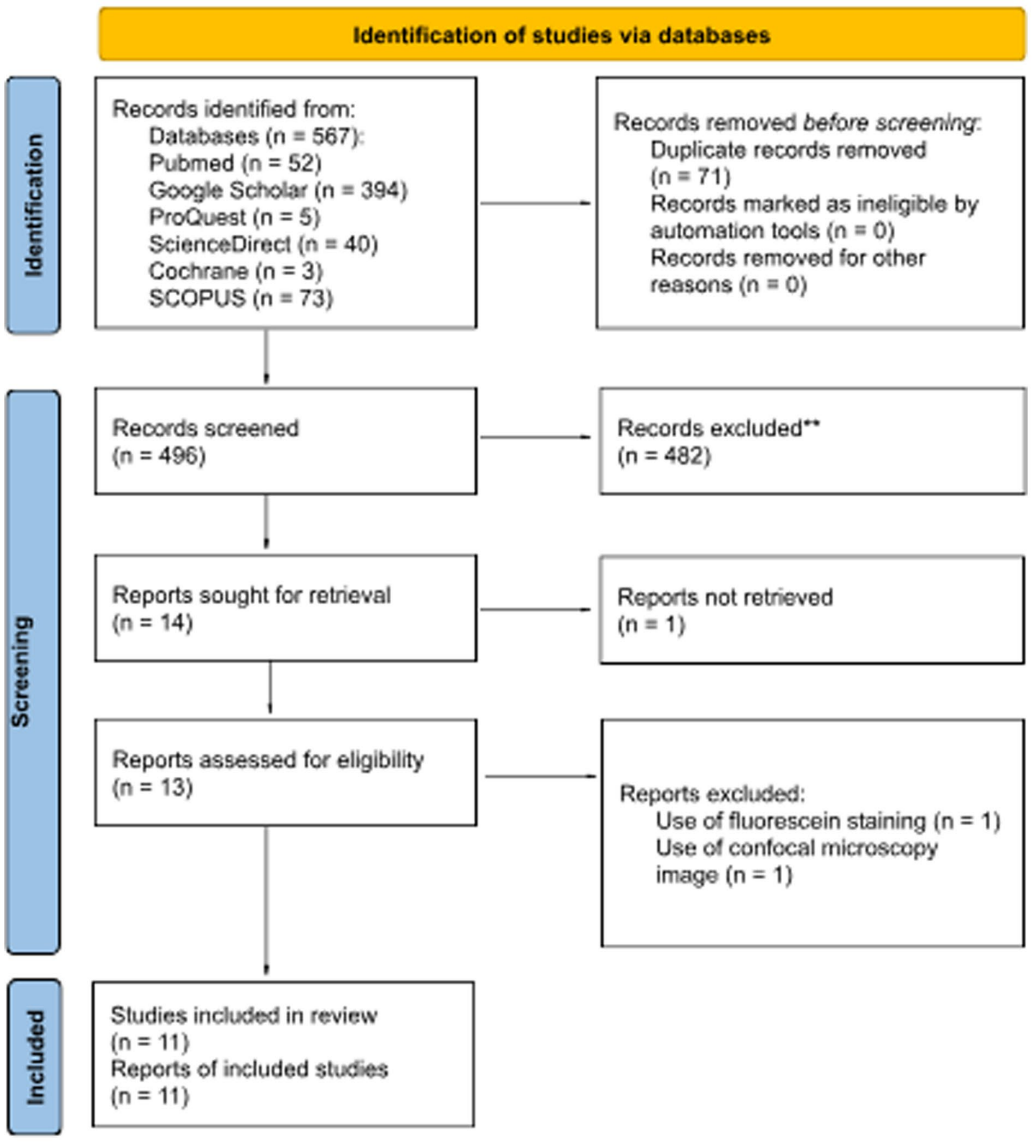
PRISMA 2020 flow diagram for updated systematic reviews that included searches of databases and registers.

Based on the search strategies previously described, articles were considered eligible to be reviewed if the article met the following criteria: (1) study on human; (2) corneal images were based on diffuse slit lamp photography of the eye; (3) validity study on DL performance. The flow chart of the literature search was described in Figure 1 based on preferred reporting items for systematic reviews and meta-analyzes (PRISMA) flow chart.

### 2.3 Internal validity assessment

Validity assessment of articles previously met our criteria using an appraisal tool for diagnostic study by the Center for Evidence-Based Medicine (CEBM), University of Oxford. Data validity, result, and applicability were assessed in all included studies. A total of 5 parameters were used, each with the results of Yes, No, or Unclear. All authors discussed discrepancies in the assessment results.

### 2.4 Data processing and extraction

The information extracted from each study includes the authors, the year the study was reported or published, baseline characteristics of subjects, number or proportion of dataset and test images, and AI algorithm name. The primary outcomes reviewed were the accuracy and classification capability of the AI/machine learning/deep learning algorithm in terms of Receiver Operator Characteristic (ROC) curve or Area Under the Curve (AUC), sensitivity, specificity, positive predictive value (PPV), negative predictive value (NPV).

### 2.5 Data analysis

The extracted data were then analyzed with the MetaXL 5.2 Software. All data on accuracy and AUC on the performance of AI on infectious keratitis diagnosis were pooled and shown in the form of forest plots and summary tables for visualization.

## 3 Results

### 3.1 Search results

A total of eleven articles were included in this review. These articles study the accuracy of various machine learning algorithms in solving the classification of keratitis based on diffuse slit lamp photography as compared with the human counterparts and cross-referenced with the respective laboratory diagnosis as the confirmatory tests.

### 3.2 Validity assessment

All eleven relevant articles were assessed for validity using standardized critical appraisal tools for diagnostic study by CEBM before proceeding to data synthesis. Validity assessment based on the CEBM Diagnostic Study Appraisal Worksheet was summarized in Table 2.

**Table 2:** Validity assessment matrix (CEBM, Oxford University^®^).

Domains	Explanatory questions	Redd et al. (2022) (21)	Zhang et al. (2022) (22)	Ghosh et al. (2021) (23)	Koyama et al. (2021) (16)	Hung et al. (2021) (18)	Sajeev et al. (2021) (24)	Li et al. (2021) (15)	Gu et al. (2020) (25)	Kuo et al. (2020) (26)	Xu et al. (2020) (8)	Saini et al. (2003) (20)
Validity	Was the diagnosis test evaluated in a representative spectrum of patients?	Y	Y	Y	Y	Y	Y	Y	Y	Y	Y	Y
	Was the reference standard applied regardless of the index test result?	Y	Y	Y	Y	Y	Y	Y	Y	Y	Y	Y
	Was there an independent, blind comparison between the index test and an appropriate reference (‘gold’) standard of diagnosis?	Y	Y	Y	Y	Y	U	U	Y	Y	Y	Y
Results	Are test characteristics presented? (Sn, Sp, PPV, NPV, AUC, accuracy)	Y	Y	Y	Y	Y	Y	Y	Y	Y	Y	Y
Applicability	Were the methods for performing the test described in sufficient detail to permit replication?	Y	Y	Y	Y	Y	Y	Y	Y	Y	Y	Y

Y, Yes; N, No; U, Unclear.

### 3.3 Included studies’ characteristics

Eleven eligible studies published between 2003 and 2022 were assessed (Table 3). Studies were conducted mainly in Asian and Australian populations, with samples acquired from the People’s Republic of China, India, Japan, Thailand, and Australia with 34,070 data from slit-lamp images.

**Table 3:** Characteristics of the included studies.

Title of Article	Author	Year	Country	Keratitis classification
Image-based differentiation of bacterial and fungal keratitis using deep convolutional neural networks	Redd et al.	2006–2015	India	BK, FK
Deep learning-based classification of infectious keratitis on slit-lamp images	Zhang et al.	June 2007 – May 2018	China	BK, FK, HSK, AK
Deep learning for discrimination between fungal keratitis and bacterial keratitis	Ghosh et al.	2012–2020	Thailand	BK, FK
Determination of probability of causative pathogen in infectious keratitis using deep learning algorithm of slit-lamp images	Koyama et al.	August 2005 – December 2020	Japan	BK, FK, HSK, AK
Using slit-lamp images for deep learning-based identification of bacterial and fungal keratitis: Model development and validation with different convolutional neural networks	Hung et al.	1 January 2010–31 December 2019	Taiwan	BK, FK
Classifying infective keratitis using a deep learning approach	Sajeev et al.	October 2018 – March 2020	Australia	BK, VK
Preventing corneal blindness caused by keratitis using artificial intelligence	Li et al.	Unspecified	China	IK, non-IK
Deep learning for identifying corneal diseases from ocular surface slit-lamp photographs	Gu et al.	April 2017 – October 2017	China	IK, non-IK
A deep learning approach in diagnosing fungal keratitis based on corneal photographs	Kuo et al.	1 June 2007–31 May 2018	Taiwan	FK, non-FK
Deep Sequential Feature Learning in Clinical Image Classification of Infectious Keratitis	Xu et al.	May 1998–2018	China	BK, FK, HSK
Neural network approach to classify infective keratitis	Saini et al.	Unspecified	India	BK, FK

AK, amebic keratitis; BK, bacterial keratitis; FK, fungal keratitis; HSK, herpes Simplex Keratitis IK, infectious keratitis; N, normal; Oth, others; PK, parasitic keratitis; VK, viral keratitis.

**Figure 2:**
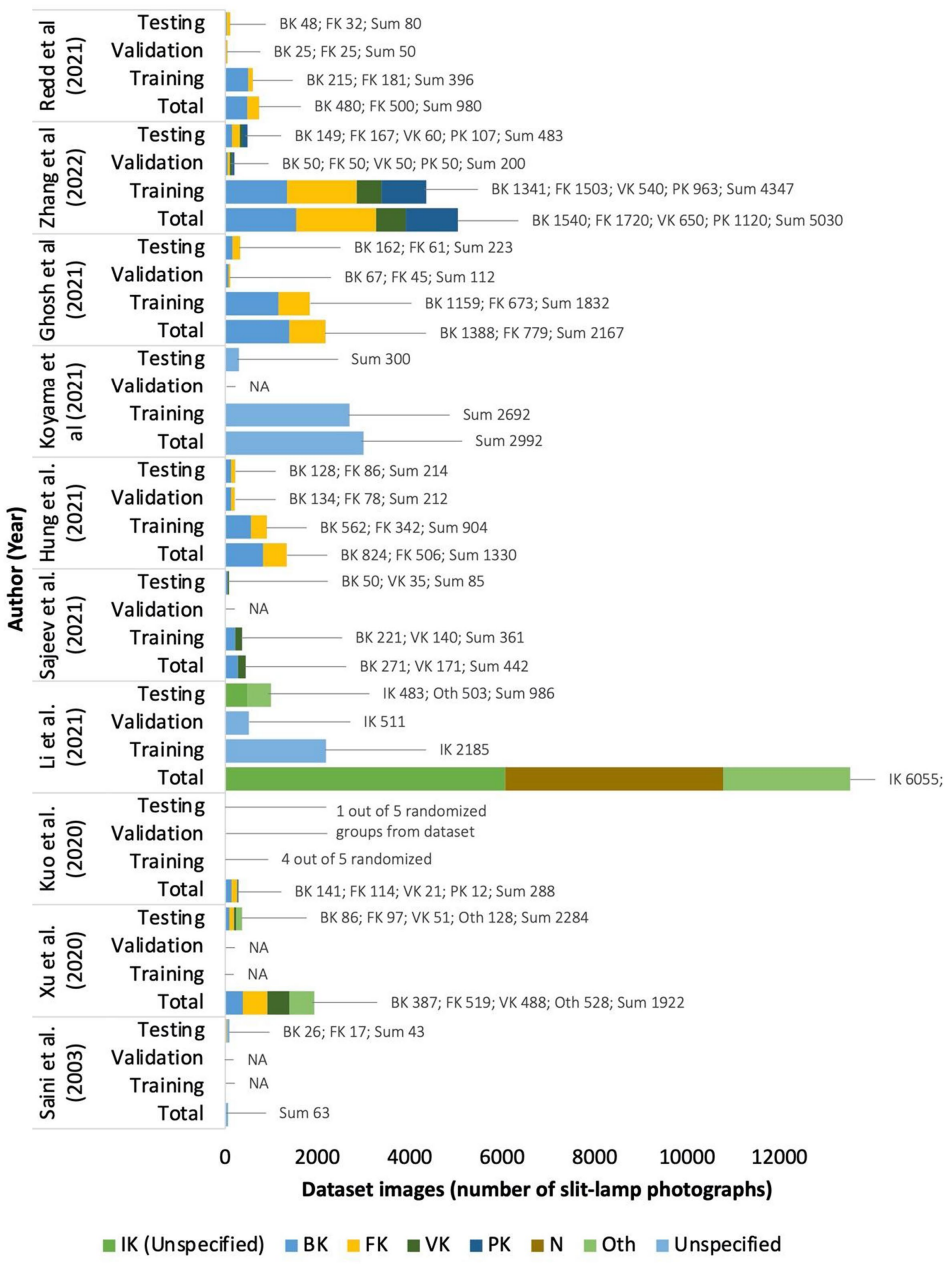
Number of dataset images of each included studies.

Nine out of the eleven studies classify IK based on two or more etiologies in which all the five studies have bacterial keratitis group; other etiologies of IK, such as fungal, viral, or parasitic are not continually assessed separately. Two studies by Li et al. (15) and Gu et al. (25) did not classify IK based on the causative microorganisms (6,055 and 845 IK images of non-specific etiologies respectively). However, these two studies compared normal cornea, infectious, and non-infectious keratitis (corneal dystrophy, degeneration, or neoplasm). No other study assessed normal corneas, with a striking amount of 6,055 [Li et al. (15)] and 870 [Gu et al. (25)] total images in their dataset. Four studies [Zhang et al. (22), Koyama et al. (16), Kuo et al. (26), Xu et al. (8)] specified viral keratitis as herpes simplex keratitis (HSK), but Sajeev et al. (24) did not specify the specific virus in the classification as the causative agent of the IK. The complete dataset classification can be seen in Figure 2 (Supplementary Table S1).

Seven of the eleven studies compare the performance of AI models with clinicians’ diagnoses made by trained ophthalmologists, corneal specialists, or non-corneal specialists. Redd et al. (21) have twelve local expert cornea specialists performing a remote interpretation of the images used in the same test set to enable direct comparison against CNN performance. Zhang et al. (22) invited three corneal specialty ophthalmologists to assess the external validation set to make a clinical diagnosis. Zhang et al. (22) invited three corneal specialty ophthalmologists to assess the external validation set to make a clinical diagnosis. Saini et al. (20) used only two corneal clinicians as a comparison. Other studies went to a greater extent to assess the human-machine comparison. Xu et al. (8) used 21 ophthalmologists trained with 120 images before the grading process, Gu et al. (25) enrolled 20 trained ophthalmologists as well as two corneal specialists to diagnose slit-lamp images of healthy and abnormal cornea conditions clinically, and Koyama et al. (16) tested 35 board-certified ophthalmologists including 16 faculty members of corneal specialist diagnosis accuracy against their AI algorithm using a diagnostic software named “KeratiTest.” Kuo et al. (26) evaluated the machine learning algorithm against two different levels of human expertise in corneal diseases: assessing a group of 3 non-cornea specialists against three corneal specialists to show if there is any difference in accuracy between the subgroups.

All eleven studies use respective laboratory examinations such as culture or direct microscopy or PCR as the reference standard for diagnosis based on the patient’s medical records in the database and prospective data. Although Sajeev et al. (24) and Gu et al. (25) did not explicitly state that the diagnoses retrieved from their database were based on a confirmatory laboratory test. The article by Saini et al. (20), however, went a step further to evaluate the correct diagnosis of IK based on the clinical improvement of the treated eye as an additional parameter of accurate diagnosis.

The established AI models presented in this review are listed in Table 4 (DenseNet121, DenseNet169, DenseNet201, EfficientNet-b0, EfficientNet-b3, EfficientNet-b5, EfficientNet-b7, Ensemble, GBDT, GoogleNet-V3, Inception-V3, InceptionResNetV2, Mobile-NetV2, ResNext101_32x8d, ResNext101_32x16d, ResNet18, ResNet34, ResNet50, ResNet101, ResNet152V2, VGG16, VGG19, Xception) were all based on convolutional neural network (CNN), a class of artificial neural network, most commonly applied to analyze visual imagery. Three studies by Sajeev et al. (24), Gu et al. (25), and Saini et al. (20) developed their algorithm to evaluate its accuracy or compare it to other established deep learning algorithms in classifying IK. Zhang et al. (22) used model blending technology and constructed KeratitisNet, their final chosen modeling method from the combination of RexNext101_32x16d and DenseNet169. The detailed training and validation process and number of images enrolled in each step were only explicitly documented in four out of ten articles [Zhang et al. (22), Hung et al. (18), Sajeev et al. (24), and Kuo et al. (26)].

**Table 4:** AUC and accuracy of different deep learning models used in the classification of IK in each study.

No	Author, year	Model	AUC	Average accuracy
1	Redd et al. (2020) (17)	MobileNet	BK vs. FK: 0.83	NA
		DenseNet	BK vs. FK: 0.83	NA
		ResNet	BK vs. FK: 0.82	NA
		VGG	BK vs. FK: 0.75	NA
		Exception	BK vs. FK: 0.75	NA
		Ensemble	BK vs. FK: 0.84	NA
2	Zhang et al. (2022) (22)	ResNet18	BK:0.82; FK:0.88; AK:0.95; HSK:0.96	68.3
		ResNet50	BK:0.84; FK:0.89; AK:0.95; HSK:0.98	72.3
		DenseNet121	BK:0.82; FK:0.87; AK:0.95; HSK:0.96	70.0
		DenseNet169	BK:0.85; FK:0.90; AK:0.95; HSK:0.98	74.0
		ResNet101_32x8d	BK:0.85; FK:0.89; AK:0.96; HSK:0.98	74.2
		ResNet101_32x16d	BK:0.84; FK:0.90; AK:0.96; HSK:0.98	75.8
		EfficientNet-b0	BK:0.85; FK:0.88; AK:0.95; HSK:0.97	72.9
		EfficientNet-b5	BK:0.82; FK:0.87; AK:0.96; HSK:0.97	72.1
		EfficientNet-b7	BK:0.83; FK:0.89; AK:0.97; HSK:0.98	74.4
		KeratitisNet	BK:0.86; FK:0.91; AK:0.96; HSK:0.98	77.8
3	Ghosh et al. (2021) (16)	VGG19	BK vs. FK: 0.86	78.0
		ResNet50	BK vs. FK: 0.60	68.0
		DenseNet121	BK vs. FK: 0.73	71.0
		Ensemble	BK vs. FK: 0.90	83.0
4	Koyama et al. (2021) (16)	ResNet-50	BK:0.82; FK:0.78; AK:0.84; HSK:0.73	88.0
		InceptionResNetV2	BK:0.87; FK:0.86; AK:0.99; HSK:0.93	87.0
5	Hung et al. (2021) (18)	DenseNet121	BK: 0.78 FK: 0.78	78.8
		DenseNet161		78.6
		DenseNet169		79.3
		DenseNet201		78.4
		EfficientNetB3		76.1
		InceptionV3		78.9
		ResNet101		80.0
		ResNet50		77.3
6	Sajeev et al. (2021) (24)	256×256 input, two CL	BK vs. VK: 0.801	75.3
		64×64 input, three CL	BK vs. VK: 0.856	81.2
7	Li et al. (2021) (15)	DenseNet121	IK: 0.988–0.990	98.0
		Inception-v3	IK: 0.976–0.989	96.8
		ResNet50	IK: 0.977–0.991	95.8
8	Gu et al. (2020) (25)	Own Model	IK: 0.930 (0.904–0.952)	NA
		InceptionV3	IK: 0.950	NA
		ResNet	IK: 0.938	NA
		DenseNet	IK: 0.954	NA
		Ensemble	IK: 0.908	NA
9	Kuo et al. (2020) (26)	Own Model	FK vs. Non-FK: 0.650	69.4
10	Xu et al. (2020) (8)	VGG16 (image-level)	Average: 0.94 BK: 0.92 FK: 0.92 HSK: 0.96	55.24
		GoogLeNet-v3 (image-level)		57.73
		DenseNet (image-level)		61.04
		VGG16 (patch-level)		45.34
		GoogLeNet-v3 (patch-level)		44.19
		DenseNet (patch-level)		59.30
		ROPs (sequence-level)		74.23
		SOPs (sequence-level)		75.14
		SOSs (sequence-level)		78.73
11	Saini et al. (2003) (20)	Own Model	NA	90.7

ROPs, Random-ordered patches; SOPs, Sequential-ordered patches; SOSs, Sequential-ordered sets.

All eleven studies used diffuse illumination slit-lamp photography of the corneal surface as the input. However, Hung et al. (18) removed the conjunctival and palpebral region to focus the observation area on the corneal area.

The number of image datasets used to train and validate the deep learning algorithm in the ten included studies varies greatly. The highest number of images for training per causative microbes’ classification is 1,690 fungal keratitis photographs in Zhang et al. (22). Study by Saini et al. (20) included 63 combination images of IK from bacterial and fungal origin. Li et al. (15) collected 6,055 IK images of unstated microorganism origin.

### 3.4 Performance of deep learning model

In the paper by Li et al. (15) and Gu et al. (25), which assessed the performance of DL algorithms to differentiate between IK and non-IK without classifying it based on the causative organism, the highest average accuracy was achieved by DenseNet121 with 98.0% accuracy, and ResNet50 achieved the lowest accuracy of 95.8%. All DL architectures in these two studies could successfully differentiate IK from non-IK with an AUC of 0.908–0.991. In classifying IK based on its respective etiologies, various available models such as InceptionV3, ResNet, and DenseNet had shown a considerable performance of 70–80% accuracy. One of the earliest DL models utilizing CNN to classify BK vs. FK was by Saini et al. (20) in 2003, which had an accuracy rate of 90.7%. The later model achieves 81.2 accuracy at maximum [BK vs. VK by Sajeev et al. (24)]. The latest study by Zhang et al. (22) compared nine model development methods and introduced KeratitisNet, demonstrating the highest average accuracy in this study. Another study by Li et al. (15) also included smartphone macro corneal photography as a dataset. It surprisingly yielded a favorable outcome, with the best algorithm reaching an AUC of 0.967 in keratitis detection. A summary of all the reviewed DL models is presented in Table 4.

### 3.5 Comparing the performance of deep learning model and human

Eight of the eleven included studies reviewed the performance of ophthalmologists of varying experience or affiliation and whether additional information or training improved visual diagnostic of IK from the available slit-lamp image data. The results showed that classifying the etiology of IK based on visual clues alone is not trivial for human experts. For instance, the study by Redd et al. (21) with AUC of 0.76 compared to 0.84 in CNN ensemble or Zhang et al. (22) with experts’ diagnostic accuracy of 31–59%. Additional patient history information, IK lesion training, and experience in cornea diseases improve human performance, as found in Li et al. (15) with increased accuracy in 6 years-experienced compared to 3 years-experienced. However, the level of accuracy was not satisfactory in almost all studies, with the highest accuracy performance being only 75% for fungal keratitis by corneal specialists in Kuo et al. (26) and did not exceed any higher than 58% even for ophthalmologists who were actively involved in teaching hospitals in Xu et al. (8). Only in a study by Li et al. (15) can a corneal specialist with 3 to 6 years of experience achieve 96% accuracy.

The AUC and accuracy in the classification of IK by the currently developed deep-learning models were summarized in Table 5. In almost all the studies, the deep-learning model was on par at a minimum compared with humans in classifying IK. In fact, in seven of the eight studies, AI models outperformed their human counterparts.

**Table 5:** Performance of ophthalmologist to classify infectious keratitis based on slit lamp images.

No	Study, year	IK Classification	Methods	Results	
				Human *vs* Human	Human *vs* DL
1	Redd et al. (2022) (21)	BK, FK	Twelve Indian cornea specialists interpreted images used in the dataset via a web-based portal	Graders AUC varied between 0.42–0.79. Human Grader Ensemble: 0.76	The CNN ensemble achieved a statistically significantly higher AUC (0.84) than the human ensemble (0.76; *p* < 0.01)
2	Zhang et al. (2022) (22)	BK, FK, HSK, AK	Three experienced cornea specialists were invited to assess the external validation data set and make a clinical diagnosis. The confusion matrix was then calculated, and diagnostic accuracy was analyzed between the models and ophthalmologists	NA	The recall rate of chosen DL in diagnosing BK, FK, AK, and HSK (70, 78, 84, 80%) was significantly higher than experts (47, 63, 31, and 59%)
3	Koyama et al. (2021) (16)	BK, FK, HSK, AK	35 board-certified ophthalmologists throughout Japan, including 16 corneal specialists of faculty members, diagnostic accuracy was assessed using a diagnostic application software named “KeratiTest” in which the AI algorithm diagnosed the single images	NA	The algorithm outperformed ophthalmologists for all types of keratitis. (AUC BK 0.82 vs. 0.58; FK 0.78 vs. 0.52; HSK 0.73 vs. 0.59; AK 0.84 vs0.59)
4	Li et al. (2021) (15)	IK, non-IK	Two corneal specialists with 3 and 6 years of experience	3 years-experience accuracy: 96.2%. 6 years-experience: accuracy: 97.3%	**Comparable to human expert with 3 years of experience.** Deep learning accuracy: 96.7%
5	Gu et al. (2020) (25)	IK, non-IK	32 ophthalmologists were trained with 90 images vs. 2 senior corneal specialists until k-value of 0.75 or more. 20 ophthalmologists qualified. Each photo was graded via face-to-face communication between two ophthalmologists. Algorithm tested against 10 ophthalmologists	NA.	**Deep-learning model outperformed the average value of all trained ophthalmologists** in sensitivity and specificity for IK and non-IK. ROC of the algorithm for each IK classification >0.910
6	Kuo et al. (2020) (26)	FK vs. non-FK	3 cornea specialists with >7 years of qualification in the specialty (26 years, 15 years, and 8 years) *VS* 3 non-cornea specialists, with comparable qualifications in clinical practice (25 years, 16 years, and 12 years). No significant difference in the average work experience (*p* = 0.8474). To grade whether the digital photos on a 28-inch liquid crystal display monitor were FK or non-FK. NCS-Oph vs. expert’s diagnosis	Average diagnostic sensitivity and NPV (negative predictive value) significantly lower in non-corneal specialists. But no significant difference in specificity and PPV. Average diagnostic accuracy: corneal spec > non-corneal specs	**The comparable average accuracy of around 70%.** Higher sensitivity than NCS-oph (52%, *p* < 0.01) but lower specificity than NCS-Oph (83%, *p* < 0.01)
7	Xu et al. (2020) (8)	BK, FK, HSK	421 ophthalmologists of different levels of expertise, experience years, affiliations trained with 120 images. 1st step is the image-only diagnosis 2nd step is the image and medical history	Teaching hospitals are far better than city hospitals/community clinics (*p* < 0.001). Attending ophthalmologist & fellow better than resident (*p* < 0.001 & *p* = 0.003) No sig. Correlation between employment duration and diagnosis accuracy	**Deep learning model is much more accurate than ophthalmologists on average, especially for HSK.** Human diagnosis using image only: Average **49.27%** (46.55% BK, 45.56% FK, 65.01% HSK). Even with medical history, only: **57.16%** (55.55% BK, 56.28% FK, 73.25% HSK). Sequence-level SOSs: Average **80.00%** (53.33% BK, 83.33% FK, 93.33% HSK) SOPs: Average **79.17%** (73.33% BK, 70.00% FK, 96.67% HSK)
8	Saini et al., 2003 (20)	BK, FK	2 corneal clinicians	NA	AI accuracy is better than human clinicians. **AI Average: 90.7%** (BK 100%, FK 76.47%). **The human average is 62.7%** (BK 61.5%, FK 64.7%)

FK, fungal keratitis; BK, bacterial keratitis; VK, viral keratitis; N, normal; IK, infectious keratitis; HSK, herpes simplex keratitis; PK, parasitic keratitis; Oth, others; NA, not available.

### 3.6 Data analysis

**Figure 3:**
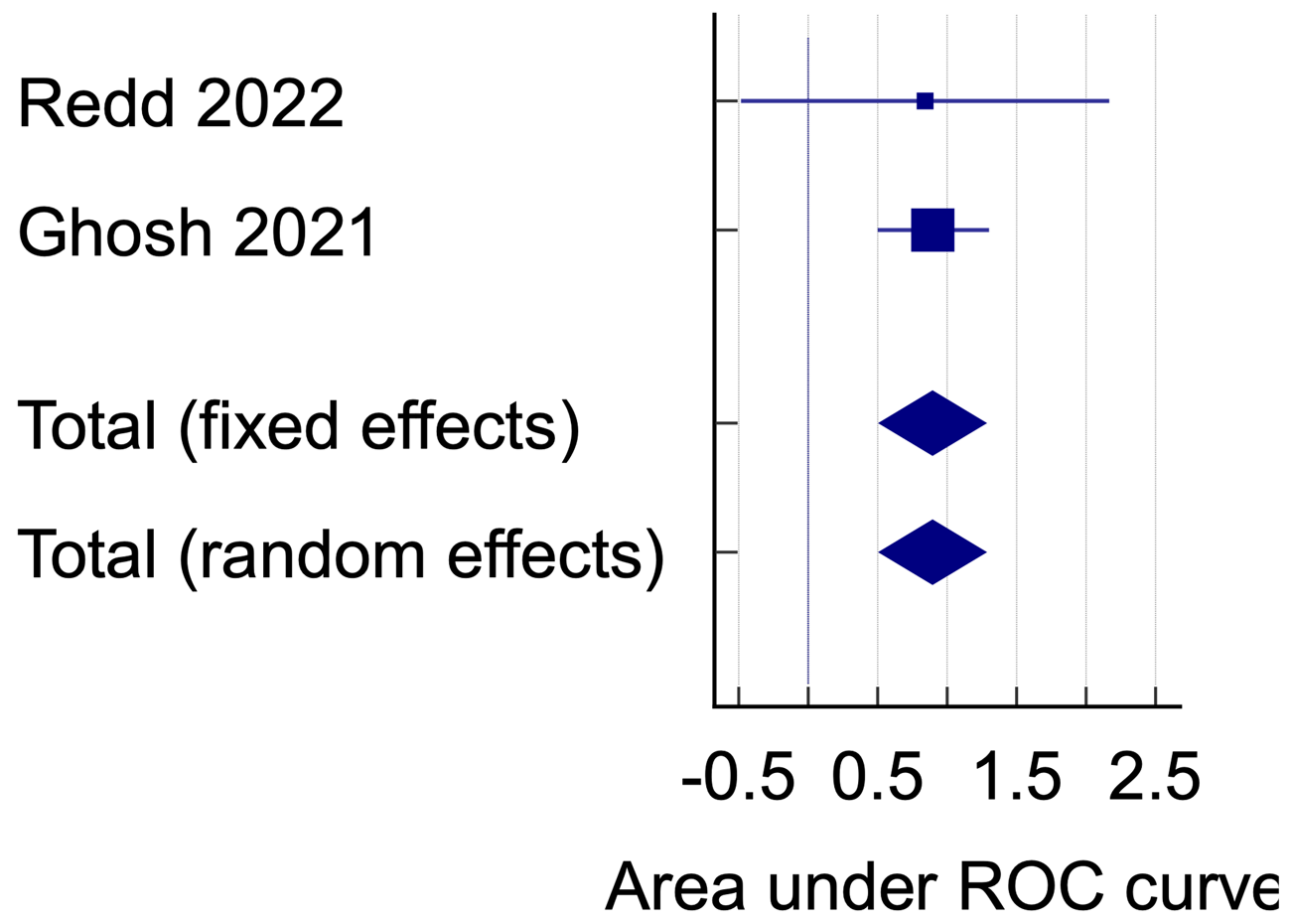
The area under the ROC curve pooled analysis on bacterial keratitis vs. fungal keratitis.

**Figure 4:**
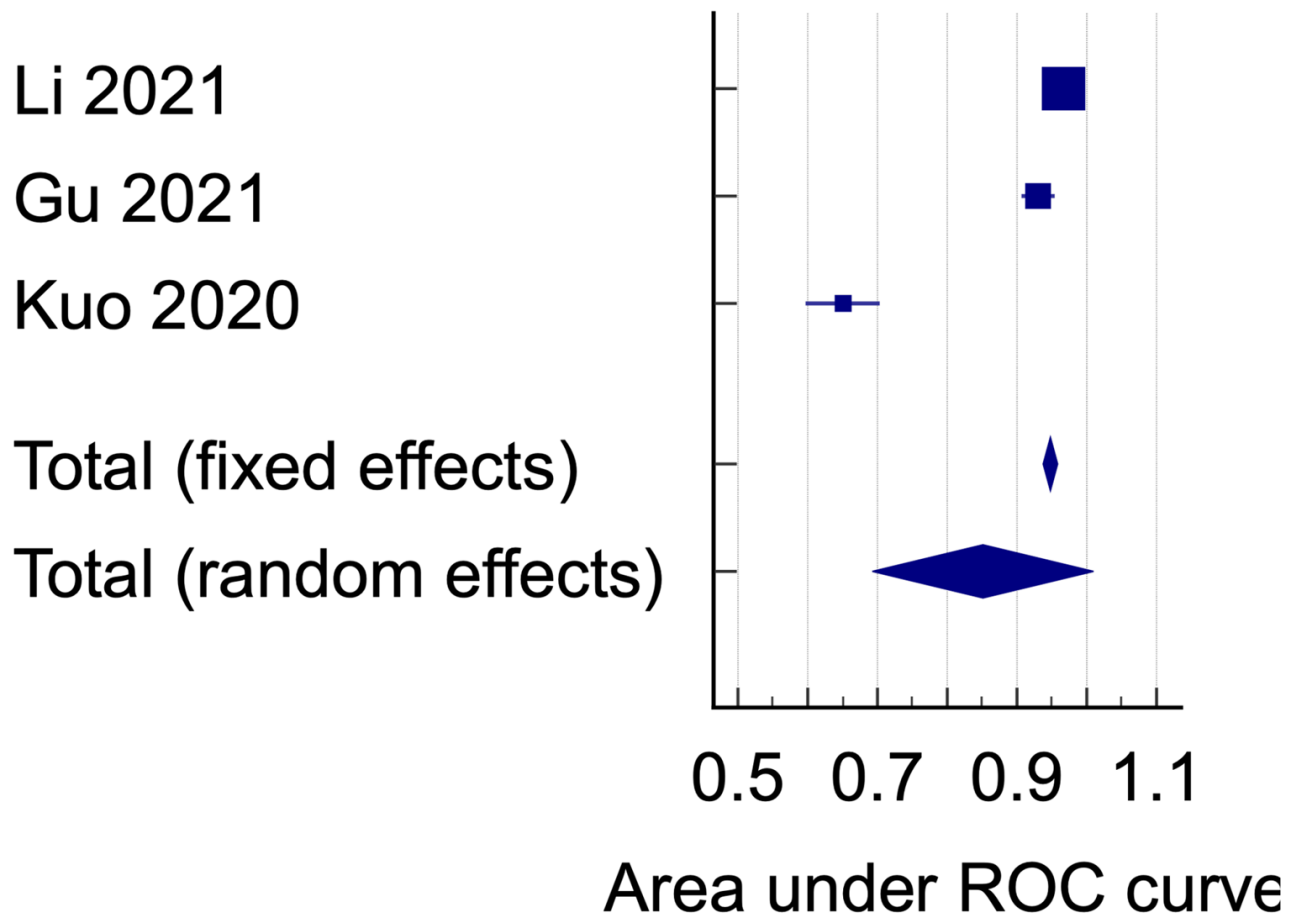
The area under ROC curve pooled analysis on infectious keratitis vs. non-infectious keratitis.

The data analysis was conducted using the data on AUC and accuracy as the performance metrics. The AUC analysis of bacterial keratitis vs. fungal keratitis used datasets from two included studies. The pooled results have shown the Receiver Operating Characteristics (ROC) curve area of 0.895 (95% CI: 0.512–1.000; *p* < 0.001), with homogenous data (Figure 2). On the contrary, the data of AUC analysis on infectious keratitis vs. non-infectious keratitis were heterogenous with I^2^ (inconsistency) of 98.51%. On the random effects analysis, the pooled AUC value was 0.851 (95% CI: 0.693–1.000) (Figures 3, 4). It has shown both studies’ relatively high performance of the artificial intelligence model.

**Figure 5:**
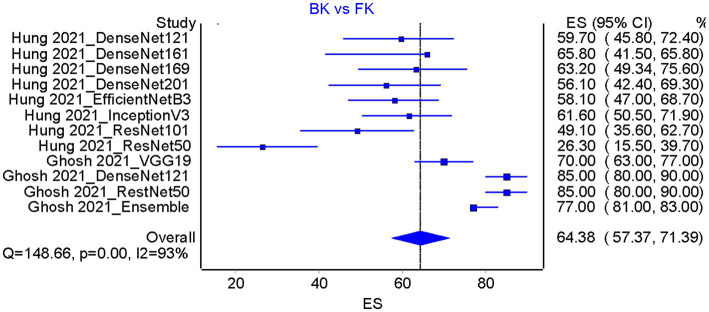
Accuracy pooled analysis on bacterial keratitis vs. fungal keratitis in two included studies [Hung et al. (18) and Ghosh et al. (23)]; ES, Effect size; CI, Confidence interval.

**Figure 6:**
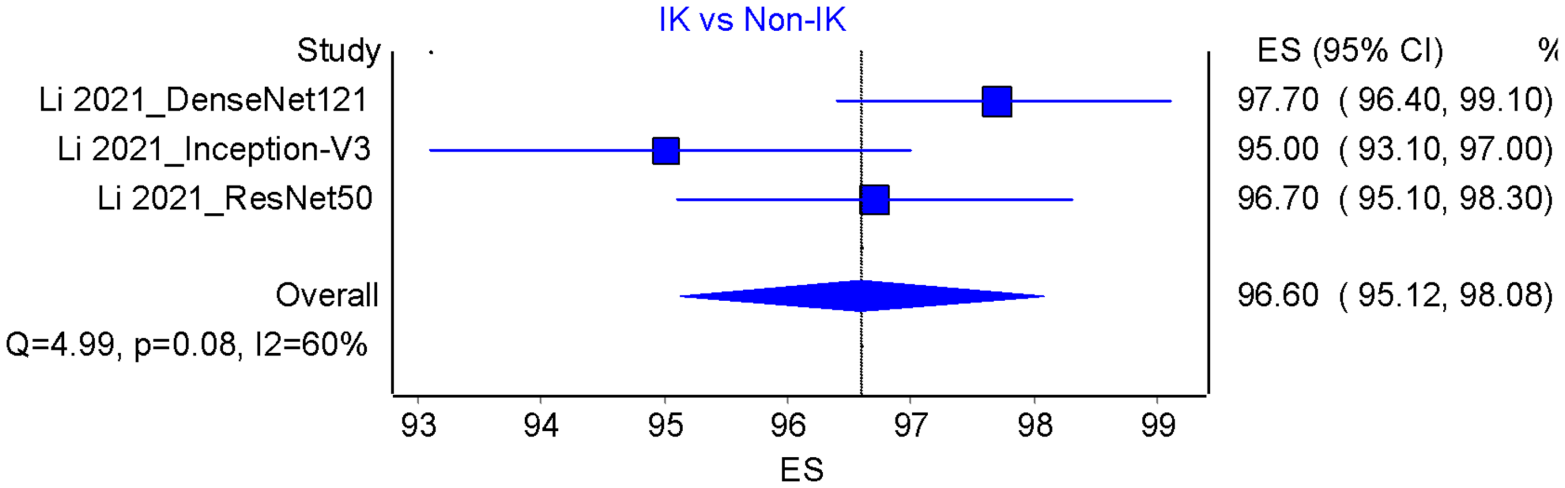
Accuracy pooled analysis on infectious vs. non-infectious keratitis; ES, Effect size; CI, Confidence interval.

Based on the results of the analysis of accuracy rate, the total accuracy value of the twelve tools tested in two studies was obtained, the study by Hung et al. (18) and Ghosh et al. (23) in differentiating between bacterial keratitis and fungal keratitis amounting to 64.38% (Figure 5). at the same time, the accuracy value of the 3 tools tested by Li et al. (15) was 96.6% (Figure 6).

## 4 Discussion

Our systematic review has shown the performance of artificial intelligence, particularly the DL model in identifying infectious keratitis. Most models perform fairly well, as seen in high accuracy and AUC analysis. The highest being DenseNet121 by Li et al. (15). Meanwhile, on the comparison between DL and human experts, DL has significantly higher accuracy than human experts in all studies, which shows a good performance of DL in IK diagnosis. On the pooled analysis, an AUC value of 0.895 on identifying bacterial keratitis vs. fungal keratitis, while AUC of 0.851 were retrieved for identifying infectious vs. non-infectious keratitis. On the accuracy analysis, pooled accuracy on BK vs. FK was 64.38%. However, the accuracy in identifying infectious vs. non-infectious keratitis was 96.6%, showing a high DL accuracy rate. The DL-based systems can learn to recognize more complex patterns and therefore have gained a particular reputation for complex applications such as ophthalmology, which require extensive image recognition tasks to identify specific clinical patterns for accurate diagnosis. Despite its growing popularity, the DL framework in ophthalmology is still in its earliest stage (27). Most DL models used to classify IK in the ten included studies used CNNs as the main framework. The CNN is the most popular neural network model for image classification. The main idea behind CNNs is to decompose image understanding into more straightforward mapping, each described by a layer in the model. For example, to recognize an apple, we teach the computer to progressively recognize lines, edges, corners, textures, and eventually apple. This notion brings about the practical advantage of requiring less feature engineering for object identification which eventually results in a more time-efficient and adequate training of the machine learning models.

### 4.1 Why did certain algorithms perform better than others?

Finding the best DL model for classifying IK is not trivial. It involves tuning various hyper-parameters that affect the performance and accuracy of the model. However, some studies have shown that more novel architectures, such as DenseNet, outperform other models in this task. DenseNet is a new neural network architecture for visual object recognition with a dense connectivity pattern. It means that each layer is connected to every other layer and reuses them in all the subsequent layers, hence the name Densely Connected Convolutional Network. This feature enhances the model by allowing it to learn more diverse and rich features from the input data. However, DenseNet is one of many possible architectures that can achieve good results on IK classification. Furthermore, models with larger capacities, e.g., deeper or broader networks, can eventually outperform smaller models when more extensive data is provided. Therefore, future works should continuously evaluate multiple architectures and compare their performance and characteristics on this problem.

### 4.2 The quest to find the one-model-to-fit-all

Even if DenseNet seemed to have the highest average accuracy, some models have better accuracy in performing a specific classification of IK. For example, in the algorithms developed by Xu et al. (8), the sequence-level classification model using ROPs (74.2%) may have a lower average accuracy than SOSs (78.7%). However, ROPs performance in diagnosing bacterial keratitis outperformed SOSs by nearly 10% (75.3% vs. 65.1%, respectively). Hence, using more than one model for each type of IK is one way to push the limit for diagnostic supremacy further. This approach is referred to as model ensemble and is an effective strategy in improving the performance of machine learning algorithms (28). Redd et al. (21) evaluated five CNNs using images of culture-proven IK and reported a higher diagnostic accuracy than cornea experts in diagnosing BK and FK (area under the ROC = 0.84 vs. 0.76). Zhang et al. (22) combine ResNext101_32x16d and DenseNet169, giving birth to the chosen final modeling method named KeratitisNet, which demonstrated the best performance and highest accuracy in diagnosing BK, FK, HSK, and AK compared with 9 other individual models. The danger of overfitting could also threaten the early development of the DL model. The pioneering model by Saini et al. (20) in 2003 correctly classified all 63 cases in the training set and performed significantly better at 90.7% accuracy, while the clinicians could only yield 62.7%. Hence, overfitting could explain how such a model could perform significantly better than the later models with more images despite containing the least number of training datasets. An overfitted function will tend to require more information and be less flexible to be applied to the real-world dataset than an optimal function. Multiple deep learning architectures with better performance are introduced every year. Given enough data, it is likely that more powerful networks will achieve better performance for the task at hand. However, the quest to find the one-model-to-fit-all may be challenging. Classifying a corneal ulcer may not be a straightforward function as the real-world application will not be as simple as bacterial and fungal alone. Duration of onset, other classes of microbial infection, mixed infection, and image quality may add to the complexity of the model.

### 4.3 Is a machine better than a human?

The overall performance of the deep learning algorithm in the included studies showed that the model outperformed the average ophthalmologist and was comparable to or better than the experience corneal specialist with years of experience in classifying IK, especially for bacterial and viral keratitis. For fungal keratitis, Xu et al. (8) reported diagnostic rates of 83.3% (higher than that of bacterial keratitis of 53.3% accuracy) using a deep sequential-level learning model with slit-lamp photographs. The accuracy of their model exceeded that of the humans by an impressive number (49.3% ± 11.9%) for over 120 test image samples. The study by Zhang et al. (22) showcases that the algorithm outperformed ophthalmologists for all 4 causative types of keratitis (BK, FK, HSK, and AK). Exceptions were found in the study by Kuo et al. (26) in which the average performance of the DL models was not as good as the overall accuracy of experts but still better than the non-corneal specialist ophthalmologist. However, even if we took a pessimistic stance towards DL classification for IK, with its sub-par performance, the DL model can still benefit primary care where most patients with IK presented. Even with a slightly lower accuracy than the experienced ophthalmologist, it will be beneficial to identify fungal keratitis since anti-fungal medications are not the empirical therapy for IK and false-negative diagnosis for FK can be devastating to the visual prognosis.

### 4.4 Limitation of DL classification

Diagnosis of less common or more complex IK such as mixed infection requires an experienced ophthalmologist or specially trained physician, or it may be missed. There is a need for further research regarding the deep learning algorithm’s ability to detect such cases which will eventually be faced in primary care settings even if it may be rare. Another critical group of classification will be the poor-quality images. Both poor-quality images and mixed infection are usually excluded in many studies with the primary intention to reduce bias or if included, will be in a small proportion. Interestingly, one paper by Li et al. (15) considered the performance of the best algorithm with poor-quality images and showed just a slightly lower AUC (0.004–0.012 difference); this number may give a hint that the performance of DL system classification even in poor-quality images will still be considered satisfactory. A study by Koyama et al. (16) also tests the algorithm’s robustness by the absence of fluorescein staining and using low-resolution web images, resulting in a minimal decrease in diagnostic efficacy. Currently, there is no minimum sample size for image dataset for a DL system to achieve a minimum acceptable performance; it is generally understood that there is no limit and the more the better, be it in the quantity or the quality of the image for training and validation of a DL architecture. In this review, Li et al. (15) study with over 6,567 images for validation, training, and testing. Using such datasets, DenseNet 121, Inception V3, and ResNet 50 could achieve accuracy between 87.1 and 98.9% in differentiating infectious keratitis vs. normal cornea and other types. This number may appear to be significant in comparison with other conventional diagnostic studies, but this number is minuscule for the development of a DL network.

To further improve DL accuracy, both the quantity and quality of the dataset input are the holy grail. Beyond just the photographs of the cornea, additional clinical data such as patient’s history, visual acuity, intraocular pressure, onset, progression, and corneal sensitivity data may further enhance the diagnostic accuracy of the deep learning model. Integration of multiple modalities may also improve the diagnostic efficacy as shown in study by Zhang et al. (22). The study by Xu et al. (8) which focused on the specific parts of infectious lesions, can yield higher accuracy than seeing the whole eye illustrate this idea well. In Xu’s study, the DenseNet model improved its performance from 60.0 to 66.3% after “voting” in patch-level classification. ROP (random-ordered patches) method can also be an alternative besides voting. However, SOS (inner-outer sequential order classification) is the most promising method to achieve the best accuracy of IK diagnosis based on the slit-lamp image alone. In addition, an AI system fed with additional clinical data or medical history might improve its performance, just like when an ophthalmologist’s image-only diagnostic improves when more patient information is integrated into the decision-making process.

### 4.5 The current environment of AI research

Together with other DL models, such as ResNet, Inception, GoogleNet, and VGGNet, they had been made as an open-source algorithm that enabled any data scientist or layperson with sufficient programming knowledge to reproduce the basic model. This inclusive, open science environment has made the development of DL rapidly bloom and hopefully remains in such a way. However, certain adjustments must be individually modified to refine the accuracy of each model. This unique fine adjustment to the base model is usually achieved by trial and error and may not be suitable for all corneal images with IK worldwide. Hence, some models may perform slightly better than others in different geographical conditions, population groups, and etiology of IK. However, the excellent accuracy of the stated model in the eleven studies comes at a price. It required a good amount of training images, in quantity and quality to yield such results. Despite the open access nature of the currently available DL models, another factor that may also hamper the replicability of the DL study is the availability of the sample images or dataset for validity and training of the DL algorithm.

### 4.6 Things that may prevent the applicability

Increasingly there has also been an open-source dataset for eye disease available to be accessed, such as *github.com* or *kaggle.com*. However, this dataset was limited by its variety, mainly sourced from Google image, and most importantly lacked the correct labeling; hence, the dataset was considered unsuitable for serious training purposes. Most models in the eleven included studies utilized well-established datasets from electronic or conventional medical records of an academic institution or hospital in the People’s Republic of China, Australia, or India. The generalizability of models reviewed for use in other populations of IK patients should be concerned, especially if used with eyes with a different anatomical structure (e.g., different ethnicities, other ocular pathology).

Furthermore, these countries had strict regulations regarding personal data protection, especially across borders, which makes replicating such studies using the same data samples impossible by individual or study group without authority. For example, The Australian legislation system has The Privacy Act (1988) which protects the handling of personal information about individuals. It includes the collection, use, storage, and disclosure of personal information in the federal public sector and the private sector. Use of Healthcare Identifiers in Australia, and access to the My Health Record system, are governed by the Healthcare Identifiers Act 2010 (HI Act) and the My Health Records Act 2012, the My Health Records Rule 2016, and the My Health Records Regulation 2012. Whereas in China, the Data Security Law (DSL) sets up a framework that classifies data collected and stored in China based on its potential impact on Chinese national security and regulates its storage and transfer depending on the data’s classification level. China’s Personal Information Protection Law (PIPL) is also in force starting August 2021, laying out ground rules around how data is collected, used, and stored. Multinational corporations (MNCs) that move personal information out of the country also will have to obtain certification on data protection from professional institutions, according to the PIPL. In the case of IK dataset, a center that would like to have a comprehensive DL-aided diagnosis for IK may have to work itself by collecting high-quality training images either from slit-lamp clinical diagnosis made by an experienced corneal specialist or through confirmatory lab tests to obtain the local rate of accuracy for implementation of DL system for IK classification since the commercially available or approved system is not available yet to this date despite its promising development. Another condition that may hamper the application of DL-assisted diagnosis of infectious keratitis is the availability of the respective treatment. In an area with a shortage of qualified diagnosticians or confirmatory laboratory facilities, scarcity of antimicrobial eye drops, be it antibiotic or anti-fungal, will usually co-exist. Unless the same effort to improve the availability of IK treatment has been achieved, the advantage of having a cheaper, faster, or more accurate diagnostic tool may not bring additional benefits to the patient.

### 4.7 Is it economical?

The fact that classification of IK is still very challenging even for trained humans with years of experience with IK lesions, developing a deep-learning model to help physicians to gain the upper hand in the war against IK for accurate diagnosis and prompt treatment can be a worthwhile effort to be made especially in countries where establishing multiple laboratory facilities is geographically or financially challenging, or where the availability of expert diagnostician is scarce and may take too long to be trained. Although there has not been any cost-effectiveness study regarding this yet, with the advent of cloud computing and the increasing availability of internet access, it may be more economically sound and efficient to set up one DL-assisted diagnostic model which can cover a wider area at a lower cost to solve the blindness problem arising from delay or mistreatment of IK in less time and with fewer resource than to invest in the high-cost and high-maintenance laboratory facility. However, cost analysis on the use of AI in ophthalmology has shown significant cost-saving nature of the implementation, such as a report by Huang et al. (29), which shows cost-effective screening of diabetic retinopathy using AI compared to traditional screening. Ruamviboonsuk et al. (30) also stated a similar report; however, the research also suggests the cost of AI implementation system in the early years might be enormous and not applicable, particularly in low or middle-income countries (22).

### 4.8 Addressing the black-box nature of DL

The “black box” nature of deep learning will still be an endless debate. It may deter skeptical future users from using deep learning-assisted diagnostic tools because it lacks an explanation despite their proven accuracy. However, this issue can be addressed with the GRAD-CAM or GRAD-CAM++ (31, 32) which shows a gradient-weighted heat map for a visual explanation of what the CNN observed as a potential corneal lesion, making cross-checking by humans possible. Although currently this system is still far from perfect, with these heatmaps, a certain level of accountability and human ‘trust’ for the machine-generated visual diagnosis will likely improve as better algorithms that adopted in future studies.

### 4.9 The limitations of this review

Firstly, even with confirmatory lab examinations, there is still room for error in corneal sampling in IK. Not all studies included in this review followed the current standard method of sample collection in IK by collecting 5–6 specimens from each affected eye using various collection devices. This collecting method increases the chance of retrieving a responsible pathogen from the corneal tissue, which has a relatively low microbial count compared to other anatomical parts. Taking multiple corneal scrapes with sharp and uncomfortable instruments is troublesome for patients and clinicians. It also increases the likelihood of contamination from inappropriate techniques, open plates, and specimen handling by non-laboratory-trained personnel (33). Not all studies adhered to the strict standard procedure of specimen collection; hence, this flaw underlies all current gold standard methods of IK diagnosis. In addition to the constraints encountered with direct microscopic pathogen identification as the gold-standard method, clinical or therapeutic diagnoses made by ophthalmologists are even more likely to compromise their accuracy, as demonstrated by some studies mentioned beforehand. Using studies with suitable sampling methods, can reduce the future dependency on a method that had an inherited low-reproducibility and low-reliability method, not to mention expensive to be maintained. Hence, in the real-world setting, using a combination of clinical diagnosis by ophthalmologists, lesion improvement by respective microbial therapy, and direct laboratory identification might still be closer to the ideal standard of IK diagnosis than just relying on one method of diagnosis.

Secondly, the ophthalmologists in each study are of a different experience. Even with the implementation of prior standardized training such as in the study by Zhang et al. (22), Gu et al. (25), and Xu et al. (8), the inter-observer variability data of the clinicians were not explicitly reviewed. Hence, comparing the overall performance of man vs. machine is difficult.

Lastly, this review only included articles or studies from online databases without manual searching. Despite the open science nature of AI-related study, the chance for unpublished material for this topic is still highly possible from the ever-growing nature of the framework and the likelihood of undisclosed research for commercial development.

## 5 Conclusion

Our study has demonstrated the promising potential of DL to diagnose and classify IK with high accuracy that competes with trained clinical experts despite the variability within the DL models architectures among the reviewed articles. However, the variability of the architectures, in addition to the nature of IK itself remain a question whether there is one most reliable model to be used in daily clinical practice. It is expected that with the continuous growth of DL advancement and future studies of DL use in IK, debilitating visual impairment or blindness due to IK can be prevented. Therefore, future studies that further analyze the accuracy of DL that also weighs in other factors are still needed.

## Author contributions

RanS and YL conceptualized the review. RanS designed the search strategies and drafted the initial manuscript. RanS, YL, and RatS critically reviewed all the included studies and revised the manuscript. AS provided expertise and added the discussion in the Artificial Intelligence or Deep Learning aspect of the study. YL and RatS approved the final manuscript and acted as the guarantor of the study. YL acts as the correspondence of the review and analysis. All authors contributed to the article and approved the submitted version.

## References

[1] Ting DSJ Ho CS Deshmukh R Said DG Dua HS. Infectious keratitis: an update on epidemiology, causative microorganisms, risk factors, and antimicrobial resistance. Eye. (2021) 35:1084–101. doi: 10.1038/s41433-020-01339-3, PMID: 33414529 PMC8102486

[2] Flaxman SR Bourne RRA Resnikoff S Ackland P Braithwaite T Cicinelli MV. Global causes of blindness and distance vision impairment 1990–2020: a systematic review and meta-analysis. Lancet Glob Health. (2017) 5:e1221–34. doi: 10.1016/S2214-109X(17)30393-5, PMID: 29032195

[3] Ting DSJ Ho CS Cairns J Elsahn A Al-Aqaba M Boswell T. 12-year analysis of incidence, microbiological profiles and in vitro antimicrobial susceptibility of infectious keratitis: the Nottingham infectious keratitis study. Br J Ophthalmol. (2021) 105:328–33. doi: 10.1136/bjophthalmol-2020-316128, PMID: 32580955 PMC7907586

[4] Collier SA Gronostaj MP MacGurn AK Cope JR Awsumb KL Yoder JS. Centers for Disease Control and Prevention (CDC). Estimated burden of keratitis--United States, 2010. MMWR Morb Mortal Wkly Rep. (2014) 63:1027–30. PMID: 25393221 PMC5779494

[5] Gopinathan U Sharma S Garg P Rao G. Review of epidemiological features, microbiological diagnosis and treatment outcome of microbial keratitis: experience of over a decade. Indian J Ophthalmol. (2009) 57:273–9. doi: 10.4103/0301-4738.53051, PMID: 19574694 PMC2712695

[6] Ting DSJ Cairns J Gopal BP Ho CS Krstic L Elsahn A. Risk factors, clinical outcomes, and prognostic factors of bacterial keratitis: the Nottingham infectious keratitis study. Front Med (Lausanne). (2021) 8:715118. doi: 10.3389/fmed.2021.715118, PMID: 34458289 PMC8385317

[7] Dalmon C Porco TC Lietman TM Prajna NV Prajna L Das MR. The clinical differentiation of bacterial and fungal keratitis: a photographic survey. Invest Ophthalmol Vis Sci. (2012) 53:1787–91. doi: 10.1167/iovs.11-8478, PMID: 22395880 PMC3342793

[8] Xu Y Kong M Xie W Duan R Fang Z Lin Y. Deep sequential feature learning in clinical image classification of infectious keratitis. Engineering. (2021) 7:1002–10. doi: 10.1016/j.eng.2020.04.012

[9] De Fauw J Ledsam JR Romera-Paredes B Nikolov S Tomasev N Blackwell S. Clinically applicable deep learning for diagnosis and referral in retinal disease. Nat Med. (2018) 24:1342–50. doi: 10.1038/s41591-018-0107-6, PMID: 30104768

[10] Ting DSW Cheung CY-L Lim G Tan GSW Quang ND Gan A. Development and validation of a deep learning system for diabetic retinopathy and related eye diseases using retinal images from multiethnic populations with diabetes. JAMA. (2017) 318:2211–23. doi: 10.1001/jama.2017.18152, PMID: 29234807 PMC5820739

[11] Al-Aswad LA Kapoor R Chu CK Walters S Gong D Garg A. Evaluation of a deep learning system for identifying glaucomatous optic neuropathy based on color fundus photographs. J Glaucoma. (2019) 28:1029–34. doi: 10.1097/IJG.0000000000001319, PMID: 31233461

[12] Gargeya R Leng T. Automated identification of diabetic retinopathy using deep learning. Ophthalmology. (2017) 124:962–9. doi: 10.1016/j.ophtha.2017.02.008, PMID: 28359545

[13] Goh JKH Cheung CY Sim SS Tan PC Tan GSW Wong TY. Retinal imaging techniques for diabetic retinopathy screening. J Diabetes Sci Technol. (2016) 10:282–94. doi: 10.1177/1932296816629491, PMID: 26830491 PMC4773981

[14] Bhaskaranand M Ramachandra C Bhat S Cuadros J Nittala MG Sadda SR. The value of automated diabetic retinopathy screening with the EyeArt system: a study of more than 100,000 consecutive encounters from people with diabetes. Diabetes Technol Ther. (2019) 21:635–43. doi: 10.1089/dia.2019.0164, PMID: 31335200 PMC6812728

[15] Li Z Jiang J Chen K Chen Q Zheng Q Liu X. Preventing corneal blindness caused by keratitis using artificial intelligence. Nat Commun. (2021) 12:3738. doi: 10.1038/s41467-021-24116-6, PMID: 34145294 PMC8213803

[16] Koyama A Miyazaki D Nakagawa Y Ayatsuka Y Miyake H Ehara F. Determination of probability of causative pathogen in infectious keratitis using deep learning algorithm of slit-lamp images. Sci Rep. (2021) 11:22642. doi: 10.1038/s41598-021-02138-w, PMID: 34811468 PMC8608802

[17] Tiwari M Piech C Baitemirova M Prajna NV Srinivasan M Lalitha P. Differentiation of active corneal infections from healed scars using deep learning. Ophthalmology. (2022) 129:139–46. doi: 10.1016/j.ophtha.2021.07.033, PMID: 34352302 PMC8792172

[18] Hung N Shih AK-Y Lin C Kuo M-T Hwang Y-S Wu W-C. Using slit-lamp images for deep learning-based identification of bacterial and fungal keratitis: model development and validation with different convolutional neural networks. Diagnostics. (2021) 11:1246. doi: 10.3390/diagnostics11071246, PMID: 34359329 PMC8307675

[19] Li W Yang Y Zhang K Long E He L Zhang L. Dense anatomical annotation of slit-lamp images improves the performance of deep learning for the diagnosis of ophthalmic disorders. Nat Biomed Eng. (2020) 4:767–77. doi: 10.1038/s41551-020-0577-y, PMID: 32572198

[20] Saini JS Jain AK Kumar S Vikal S Pankaj S Singh S. Neural network approach to classify infective keratitis. Curr Eye Res. (2003) 27:111–6. doi: 10.1076/ceyr.27.2.111.15949 14632163

[21] Redd TK Prajna NV Srinivasan M Lalitha P Krishnan T Rajaraman R. Image-based differentiation of bacterial and fungal keratitis using deep convolutional neural networks. Ophthalmol Sci. (2022) 2:100119. doi: 10.1016/j.xops.2022.100119, PMID: 36249698 PMC9560557

[22] Zhang Z Wang H Wang S Wei Z Zhang Y Wang Z. Deep learning-based classification of infectious keratitis on slit-lamp images. Ther Adv Chronic Dis. (2022) 13:204062232211360. doi: 10.1177/20406223221136071, PMID: 36407021 PMC9666706

[23] Ghosh AK Thammasudjarit R Jongkhajornpong P Attia J Thakkinstian A. Deep learning for discrimination between fungal keratitis and bacterial keratitis: DeepKeratitis. Cornea. (2022) 41:616–22. doi: 10.1097/ICO.0000000000002830 34581296 PMC8969839

[24] Sajeev S Prem SM (2021). Classifying infective keratitis using a deep learning approach. 2021 Australasian computer science week multiconference. New York, NY, USA: ACM.

[25] Gu H Guo Y Gu L Wei A Xie S Ye Z. Deep learning for identifying corneal diseases from ocular surface slit-lamp photographs. Sci Rep. (2020) 10:17851. doi: 10.1038/s41598-020-75027-3, PMID: 33082530 PMC7576153

[26] Kuo M-T Hsu BW-Y Yin Y-K Fang P-C Lai H-Y Chen A. A deep learning approach in diagnosing fungal keratitis based on corneal photographs. Sci Rep. (2020) 10:14424. doi: 10.1038/s41598-020-71425-9, PMID: 32879364 PMC7468230

[27] Tahvildari M Singh RB Saeed HN. Application of artificial intelligence in the diagnosis and Management of Corneal Diseases. Semin Ophthalmol. (2021) 36:641–8. doi: 10.1080/08820538.2021.1893763 33689543

[28] Dietterich TG. “Ensemble methods in machine learning.” (2000). p. 1–15 Springer, Berlin, Heidelberg

[29] Huang X-M Yang B-F Zheng W-L Liu Q Xiao F Ouyang P-W. Cost-effectiveness of artificial intelligence screening for diabetic retinopathy in rural China. BMC Health Serv Res. (2022) 22:260. doi: 10.1186/s12913-022-07655-6, PMID: 35216586 PMC8881835

[30] Ruamviboonsuk P Chantra S Seresirikachorn K Ruamviboonsuk V Sangroongruangsri S. Economic evaluations of artificial intelligence in ophthalmology. Asia Pac J Ophthalmol. (2021) 10:307–16. doi: 10.1097/APO.0000000000000403, PMID: 34261102

[31] Selvaraju RR Cogswell M Das A Vedantam R Parikh D Batra D. Grad-CAM: visual explanations from deep networks via gradient-based localization. Int J Comput Vis. (2019) 128:336–59. doi: 10.1007/s11263-019-01228-7

[32] Chattopadhay A Sarkar A Howlader P Balasubramanian VN. Grad-CAM++: generalized gradient-based visual explanations for deep convolutional networks. 2018 IEEE winter conference on applications of computer vision (WACV). IEEE. (2018).

[33] Pakzad-Vaezi K Levasseur SD Schendel S Mark S Mathias R Roscoe D. The corneal ulcer one-touch study: a simplified microbiological specimen collection method. Am J Ophthalmol. (2015) 159:37–43.e1. doi: 10.1016/j.ajo.2014.09.021, PMID: 25244977

