# Supplementary material for: Accuracy of artificial intelligence model for infectious keratitis classification: a systematic review and meta-analysis
Source: Front Public Health. 2023 Nov 24;11:1239231. doi: 10.3389/fpubh.2023.1239231 (PMC10704127; doi:10.3389/fpubh.2023.1239231)
Supplement: Supplementary file 1 [file Table_1.docx]

Supplementary Material

**Accuracy of Artificial Intelligence Model for Infectious Keratitis Classification: A Systematic Review and Meta-Analysis**

# Supplemental Tables

**Supplementary Table 1**: Characteristics of the included studies

| **No** | **Author (year)** | **Keratitis Classification** | **Dataset images (number of slit-lamp photographs)** | | | | | | | | | | | | | | | | | | |
| --- | --- | --- | --- | --- | --- | --- | --- | --- | --- | --- | --- | --- | --- | --- | --- | --- | --- | --- | --- | --- | --- |
|  |  |  | **Total** | | | | | | **Training** | | | | **Validation** | | | | **Testing** | | | | |
|  |  |  | **BK** | **FK** | **VK** | **PK** | **N** | **Oth** | **BK** | **FK** | **VK** | **PK** | **BK** | **FK** | **VK** | **PK** | **BK** | **FK** | **VK** | **PK** | **Oth** |
| 1 | Redd et al.  (2022) | BK, FK | 980 | | | | | | 396 | | | | 50 | | | | 80 | | | | |
|  |  |  | 480 | 500 | NA | NA | NA | NA | 215 | 181 | NA | NA | 25 | 25 | NA | NA | 48 | 32 | NA | NA | NA |
| 2 | Zhang et al.  (2022) | BK, FK,  HSK, AK | 5030 | | | | | | 4347 | | | | 200 | | | | 483 | | | | |
|  |  |  | 1540 | 1720 | 650 | 1120 | NA | NA | 1341 | 1503 | 540 | 963 | 50 | 50 | 50 | 50 | 149 | 167 | 60 | 107 | NA |
| 3 | Ghosh et al.  (2021) | BK, FK | 2167 | | | | | | 1832 | | | | 112 | | | | 223 | | | | |
|  |  |  | 1388 | 779 | NA | NA | NA | NA | 1159 | 673 | NA | NA | 67 | 45 | NA | NA | 162 | 61 | NA | NA | NA |
| 4 | Koyama et al.  (2021) | BK, FK,  HSK, AK | 2992 | | | | | | 2692 | | | | NA | | | | 300 | | | | |
|  |  |  | NA | | | | | | NA | | | |  |  |  |  | NA | | | | |
| 5 | Hung et al.  (2021) | BK, FK | 1330 | | | | | | 562 | 342 | NA | NA | 134 | 78 | NA | Na | 128 | 86 | NA | NA | NA |
|  |  |  | 824 | 506 | NA | NA | NA | NA |  |  |  |  |  |  |  |  |  |  |  |  |  |
| 6 | Sajeev et al.  (2021) | BK, VK | 442 | | | | | | 221 | NA | 140 | NA | NA | | | | 50 | NA | 35 | NA | NA |
|  |  |  | 271 | NA | 171 | NA | NA | NA |  |  |  |  |  |  |  |  |  |  |  |  |  |
| 7 | Li et al.  (2021) | IK vs non-IK | 13557 | | | | | | 2185 | | | | 511 | | | | 483 | | | | 503 |
|  |  |  | 6055 | | | | 4725 | 2777 |  |  |  |  |  |  |  |  |  |  |  |  |  |
| 8 | Gu et al.  (2020) | IK vs non-IK | 5325 | | | | | | NA | | | | NA | | | | 86 | | | | 424 |
|  |  |  | 845 | | | | 879 | 3610 |  |  |  |  |  |  |  |  |  |  |  |  |  |
| 9 | Kuo et al.  (2020) | FK vs non-FK | 288 | | | | | | 4 out of 5 randomised | | | | 1 out of 5 randomised groups from dataset | | | | | | | | |
|  |  |  | 141 | 114 | 21 | 12 | NA | NA |  |  |  |  |  |  |  |  |  |  |  |  |  |
| 10 | Xu et al.  (2020) | BK, FK, HSK | 1922 | | | | | | NA | | | | NA | | | | 86 | 97 | 51 | NA | 128 |
|  |  |  | 387 | 519 | 488 | NA | NA | 528 |  |  |  |  |  |  |  |  |  |  |  |  |  |
| 11 | Saini et al.  (2003) | BK, FK | 63 | | | | | | NA | | | | NA | | | | 26 | 17 | NA | NA | NA |
|  |  |  | NA | NA | NA | NA | NA | NA |  |  |  |  |  |  |  |  |  |  |  |  |  |

Notes: FK=fungal keratitis; BK=
